# Clustering patterns of metabolic syndrome: A cross-sectional study in children and adolescents in Kyiv

**DOI:** 10.3389/fped.2022.972975

**Published:** 2022-11-07

**Authors:** Maiia H. Aliusef, Ganna V. Gnyloskurenko, Alina V. Churylina, Inga O. Mityuryayeva

**Affiliations:** Department of Pediatrics, Bogomolets National Medical University, Kyiv, Ukraine

**Keywords:** cluster analysis, insulin resisitance, insulin-secreting cells, metabolic syndrome, waist circumference

## Abstract

**Objective:**

The aim: to identify subgroups by cluster analysis according parameters: original homeostatic model of insulin resistance (HOMA-1 IR), updated computer model of insulin resistance (HOMA-2 IR), β-cell function (%B) and insulin sensitivity (%S) for the prognosis of different variants of metabolic syndrome in children for more individualized treatment selection.

**Patients and methods:**

The observational cross-sectional study on 75 children aged from 10 to 17 with metabolic syndrome according to the International Diabetes Federation criteria was conducted at the Cardiology Department of Children's Clinical Hospital No.6 in Kyiv. HOMA-1 IR was calculated as follows: fasting insulin (µIU/ml) × fasting glucose (mmol/L)/22.5. HOMA-2 IR with %B and %S were calculated according to the computer model in [http://www.dtu.ox.ac.uk]. All biochemical analysis were carried out using Cobas 6000 analyzer and Roche Diagnostics (Switzerland). The statistical analysis was performed using STATISTICA 7.0 and Easy R. The hierarchical method Ward was used for cluster analysis according the parameters: HOMA-1 IR, HOMA-2 IR, %B and %S.

**Results:**

Four clusters were identified from the dendrogram, which could predict four variants in the course of metabolic syndrome such that children in cluster 1 would have the worst values of the studied parameters and those in cluster 4 – the best. It was found that HOMA-1 IR was much higher in cluster 1 (6.32 ± 0.66) than in cluster 4 (2.19 ± 0.13). HOMA-2 IR was also much higher in cluster 1 (3.80 ± 0.34) than in cluster 4 (1.31 ± 0.06). By the analysis of variance using Scheffe's multiple comparison method, a statistically significant difference was obtained between the laboratory parameters among the subgroups: HOMA-1 IR (*p* < 0,001), glucose (*p* < 0.001), insulin (*p* < 0,001), HOMA-2 IR (*p* < 0.001), %B (*p* < 0.001), %S (*p* < 0.001), TG **(***p* = 0.005) and VLDL-C (*p* = 0.002).

**Conclusions:**

A cluster analysis revealed that the first two subgroups of children had the worst insulin resistance and lipid profile parameters. It was found positive correlation between HOMA-1 IR, HOMA-2 IR, %B and %S with lipid metabolism parameters TG and VLDL-C and negative correlation between %B and HDL-C in children with metabolic syndrome (MetS).The risk of getting a high TG result in the blood analysis in children with MetS was significantly dependent with the HOMA-2 IR >2.26.

## Introduction

The prevalence of metabolic syndrome in children varies widely according to different sources (1) and various diagnostic methods (2) This is due to not only the differences between nations and races, but also to lack of a unified diagnostic consensus in the pediatric population. Metabolic syndrome (MetS) represents a combination of multiple disorders including central or abdominal obesity, elevated blood pressure, dislipidemia, type 2 diabetes mellitus or impaired glucose tolerance. According to the guidelines established by the International Diabetes Federation (IDF), MetS is diagnosed in children from the age of 10 years with the following criteria: abdominal obesity plus any two of four factors including high blood pressure, high triglyceride, low high-density lipoprotein cholesterol, high fasting plasma glucose (3).

The definition assumes that MetS is heterogeneous disease and one individual may have abdominal obesity, low HDL-C and high fasting plasma glucose, while another one has abdominal obesity, high blood pressure and high TG. Therefore, it is important to cluster patients based on one of the etiological mechanisms of the disease. One of the main long-established pathogenetic underpinnings for the development of the MetS is insulin resistance (IR) (4, 5). Many methods to measure IR have been published in the past. The most commonly used is the homeostatic model of insulin resistance (HOMA-IR), which is based on fasting insulin and glucose values. The original HOMA-1 IR model was described in 1985. Reduced *β*-cell function was modeled by changing the *β*-cell response concentration of plasma glucose. An updated HOMA (HOMA-2 IR) is the computer model in 1996 was calibrated to obtain *β*-cell function (%B) and insulin sensitivity (%S), given that a level of 100% is normal and IR of 1 (6). HOMA2-IR model has nonlinear solutions and these should be used compared with the HOMA1-IR minimal model. Also, HOMA-2 IR model accounts for variations in hepatic and peripheral glucose tolerance and reduced peripheral glucose uptake stimulated by glucose. Previously, it was thought that insulin resistance causes an increase in plasma glucose levels, which contribute to the need for pancreatic insulin-secreting cells (*β*-cells) to produce and secrete more insulin. Chronic exposure to elevated glucose leads to *β*-cell dysfunction and cell deathcausing diabetes (7).

However, recently the theory that insulin resistance precedes beta cell dysfunction has been challenged and it is believed that beta cell hyperfunction leads subsequently to IR (8).

Therefore, appreciable destruction of *β*-cell, may occur often before the impaired glucose tolerance or persistent elevation of fasting glucose. It is also known that excessive cholesterol accumulation in *β*-cells can cause lipotoxicity and reduce insulin secretion, causing a reduction in the number of *β*-cells (9). Early studies have shown that people with hyperglycaemia have a more severely impaired lipid profile. Studies demonstrate that LDL-C inhibits glucose-stimulated insulin secretion and *β*-cell proliferation *via* LDL receptor mechanisms. Also, high levels of LDL-C (>6 mmol/L) cause *β*-cell apoptosis (10). Given the pathogenetic links between the processes outlined above, we suggested that beta cell activity and insulin sensitivity may be prognostic criteria for severity of the MetS. Therefore, the aim of our study was to identify subgroups of children with metabolic syndrome based on these indicators.

## Materials and methods

The observational cross-sectional study on 75 children aged 10–17 with metabolic syndrome (MetS) was conducted at the Cardiology Department of Children's Clinical Hospital No.6 in Kyiv. The sample size was calculated using the the package G*Power 3.1.9.7 (11). under the assumption of odds ratio =5 with frequency of qualitative characteristics −0.5 (50%) and a critical significance level of 0.05 and power −0.8. Calculation for quantitative characteristics was done assuming odds ratio =5 with a change of 0.5*σ* with a critical significance level of 0.05, power −0.8. Inclusion criteria: all children who came to the clinic with the complaint of being overweight and who were diagnosed with MetS based on the IDF criteria: which include abdominal obesity plus two or more factors including blood pressure ≥130/85 mmHg, triglyceride level ≥1.7 mmol/L, high-density lipoprotein cholesterol <1.03 mmol/L, fasting plasma glucose ≥5.6 mmol/L. Exclusion criteria: patients with metabolic syndrome, associated with genetic syndromes. All anthropometric measurements were performed immediately upon the patient's admission to the department. Body mass index and waist circumference according to growth charts were determined at all children (WC). BMI was assessed according to WHO growth charts. Abdominal obesity was established by measuring the WC ≥90 percentile for age and sex-specific. As there are no national WC charts, it was used the British references in adolescents as they reflect the patterns of WC in Caucasian children (12). 24-h ambulatory blood pressure monitoring was performed using ABM-04 («Meditech», Hungary) (13, 14). There were determined the parameters of the lipid profile which include total cholesterol (TC), triglycerides (TG), high-density lipoproteins (HDL-C), low-density lipoproteins (LDL-C), very low density lipoproteins (VLDL-C), atherogenic coefficient (AC). HOMA-1 IR was calculated using the formula: fasting insulin (µIU/ml) × fasting glucose (mmol/L)/22.5. HOMA-2 IR with beta-cell function (%B) and insulin sensitivity (%S) were calculated according to the computer model in [http://www.dtu.ox.ac.uk]. All biochemical analysis were carried out by enzymatic colorimetric method of Cobas 6000, Roche Diagnostics (Switzerland) analyzer and test system. No patient was excluded at the end of the study. The hierarchical method Ward was performed for cluster analysis using the statistical package STATISTICA 7.0, which aim to minimize the main variation within groups based on Euclidean distances. A non-parametric Spearman rank correlation test was used for the correlation analysis. The critical level of significance was estimated to be equal to or less than 0.05. In order to predict the binary initial variable under the influence of the factor variables, a logistic regression model was used. ROC-curve analysis was used to assess the quality of the logistic regression model. The statistical analysis was performed using EZR (Easy R) version 1.54 **(**December 24, 2020).

There may be some possible limitations in this study. This research is a pilot study and it is limited to the city of Kyiv. Also, the IDF classification limits the diagnosis of MetS to children from the age of 10 years, so the results of this article cannot be extrapolated to younger children. For children from 2 to 11 years, the IDEFICS classification should be used to diagnose MetS (15).

## Results

**Figure 1:**
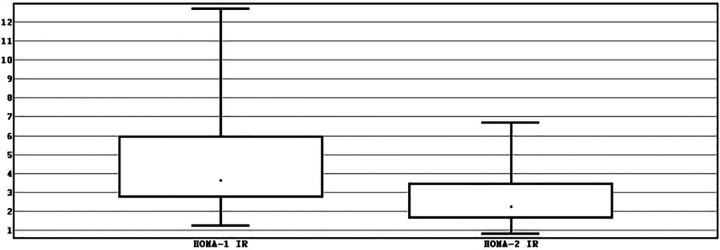
Interval evaluation of HOMA-1 IR and HOMA-2 IR.

The sample consisted of more males −60% (*n* = 60) than females −20% (*n* = 15) with a median of age of 15 years [interquartile range (IQR) 14–16]. Median BMI was of 28.73 kg/m^2^ (IQR 26.85–31.74). Median WC – 91.5 cm (IQR 87.00–96.00). Median HOMA-1 IR was significantly higher −3.65 (IQR 2.75–5.96) than median HOMA-2 IR – 2.26 (IQR 1.64–3.46), *p* < 0.001 (Figure 1).

Median %B – 198.95 (IQR 142.35–253.65), median %S – 44.7 (IQR 29.05–61.4).

There was no significant difference between the median BMI of boys (28.71 ± 0.59), and girls (30.43 ± 1,47), *p* = 0.344. The WC was significantly higher in boys (92 ± 1.79) compared to girls (86 ± 1.52). HOMA-1 IR did not differ between the groups: 3.54 ± 0.37 and 5.99 ± 0.96 respectively, *p* = 0.085. HOMA-2 IR had a gender difference with score for males –2.17 ± 0.19 and females −3.38 ± 0.502, *p* = 0.049. Glucose levels did not differ between genders −4.76 ± 0.09 and 4.99 ± 0.18, *p* = 0.269. The median insulin in boys (17.5 ± 1.702) was significantly lower than girls (27 ± 4.56), *p* = 0.045. Accordingly, %S showed higher values in boys (46.05 ± 3.53) than girls (29.6 ± 6.87) with *p* = 0.049. The median %B did not differ significantly between the males – 186.3 ± 13.22 and females – 224.1 ± 36.51 with *p* = 0.206.

**Figure 2:**
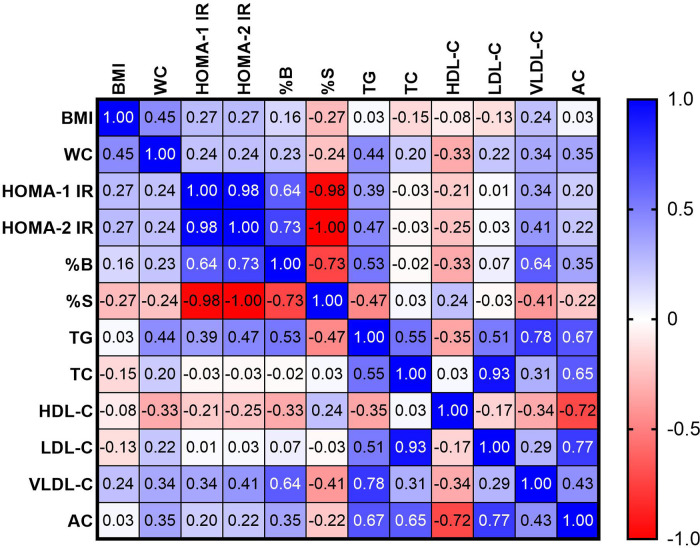
Heat map correlation between parameters of insulin resistance and parameters of lipid profile.

In order to assess the relationship between the parameters of insulin resistance and lipid metabolism, a correlation analysis was performed. The results of the study are summarized in the heat map (Figure 2). The positive correlations were found between TG and HOMA-1 IR (*r* = 0.39), HOMA-2 IR (*r* = 0.47) and %B (*r* = 0.53) and negative correlation between TG and %S (*r* = −0.47), *p* < 0.05. VLDL-C was positively correlated with HOMA-1 IR (*r* = 0.34), HOMA-2 IR (*r* = 0.41) and %B (*r* = 0.64) and negatively with %S (*r* = −0.41). The negative correlation was observed between HDL-C and %B with *r* = 0.33 (*p* < 0.05). The positive correlation was found between AC and %B (*r* = 0.35), *p* < 0.05.

**Figure 3:**
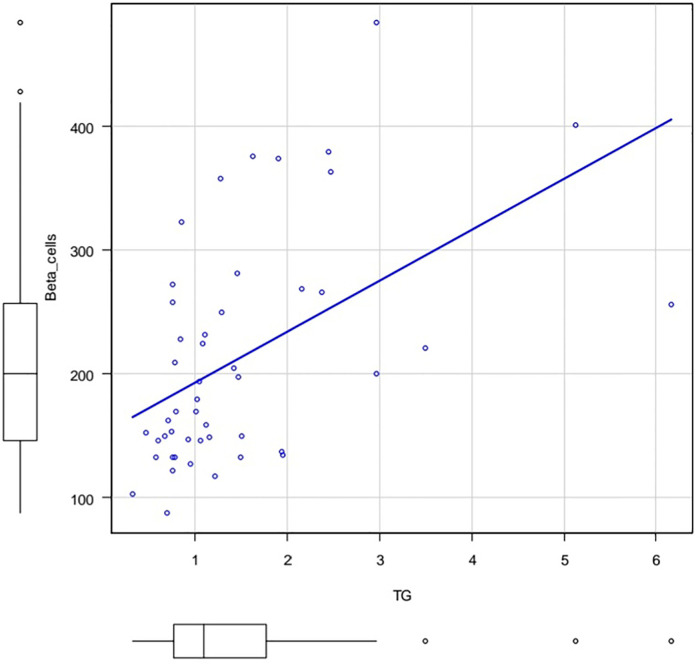
Correlation field in coordinates: TG (X axis) and %B (Y axis). Spearman's rank correlation coefficient 0.53 *p*.value = 0.000105.

**Figure 4:**
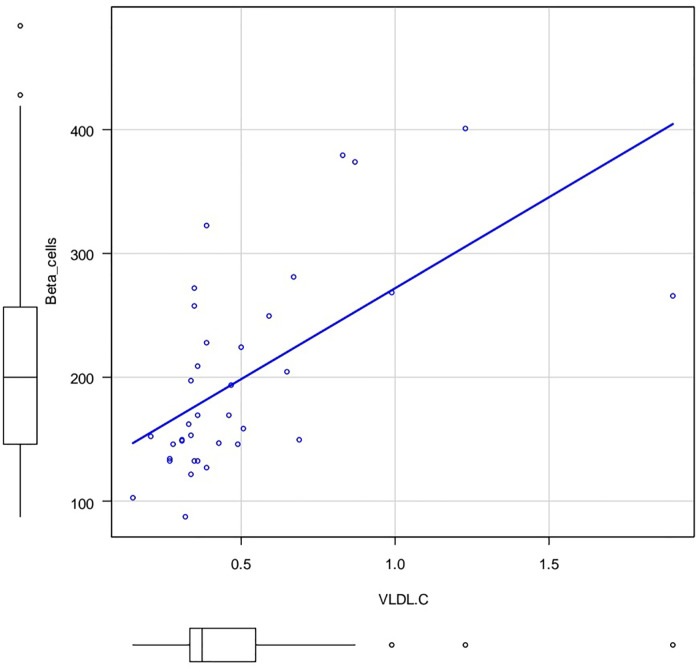
Correlation field in coordinates: VLDL-C (X axis) and %B (Y axis). Spearman's rank correlation coefficient 0.641 *p*.value = 0.0000257.

The Figures 3, 4 show the correlation field of a pair of indices, where a correlation of medium strength was detected (Figures 3, 4).

**Figure 5:**
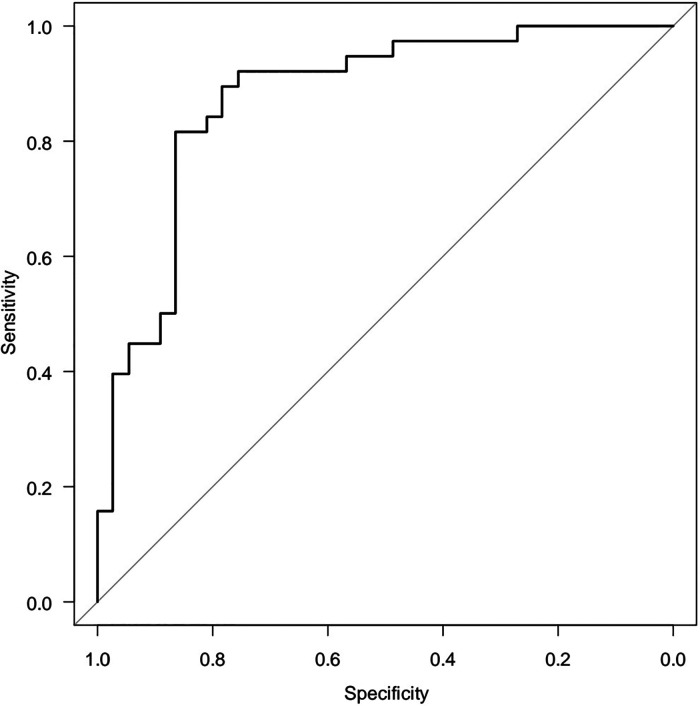
ROC-curve two-factor logistic regression model (AUC = 0.876 95% CI 0.794–0.957).

Since the correlation analysis only answers the question of the presence of the relationship between the parameters, but not the cause-and-effect relationship, It was conducted a logistic regression model, where Y is a binary initial sign of the influence of factor variables: X_1_, X_2_,…X_m_. Hence, for HOMA-2 IR there are no stabilized reference values, we suggested HOMA-2 IR ≤2.26 as the normal range and >2.26 as high. The relationship between normal and high ranges HOMA-2 IR (Y) with age (X_1_) and beta cell function (X_2_) was analysed. A two-factor model revealed a relationship between HOMA-2 IR and beta cell function [OR = 1.020 (95%CI 1.010–1.040) *p* = 0.0000588] and age [OR = 0.663 (95%CI 0.463–0.951) *p* = 0.256]. The area under the ROC (receiver operating characteristics) curve, AUC = 0.876 (95%CI 0.794–0.957), is significantly (*p* < 0.05) greater than 0.5, indicating the adequacy of the constructed model. The quality of the model was assessed as “very good” (Figure 5).

**Figure 6:**
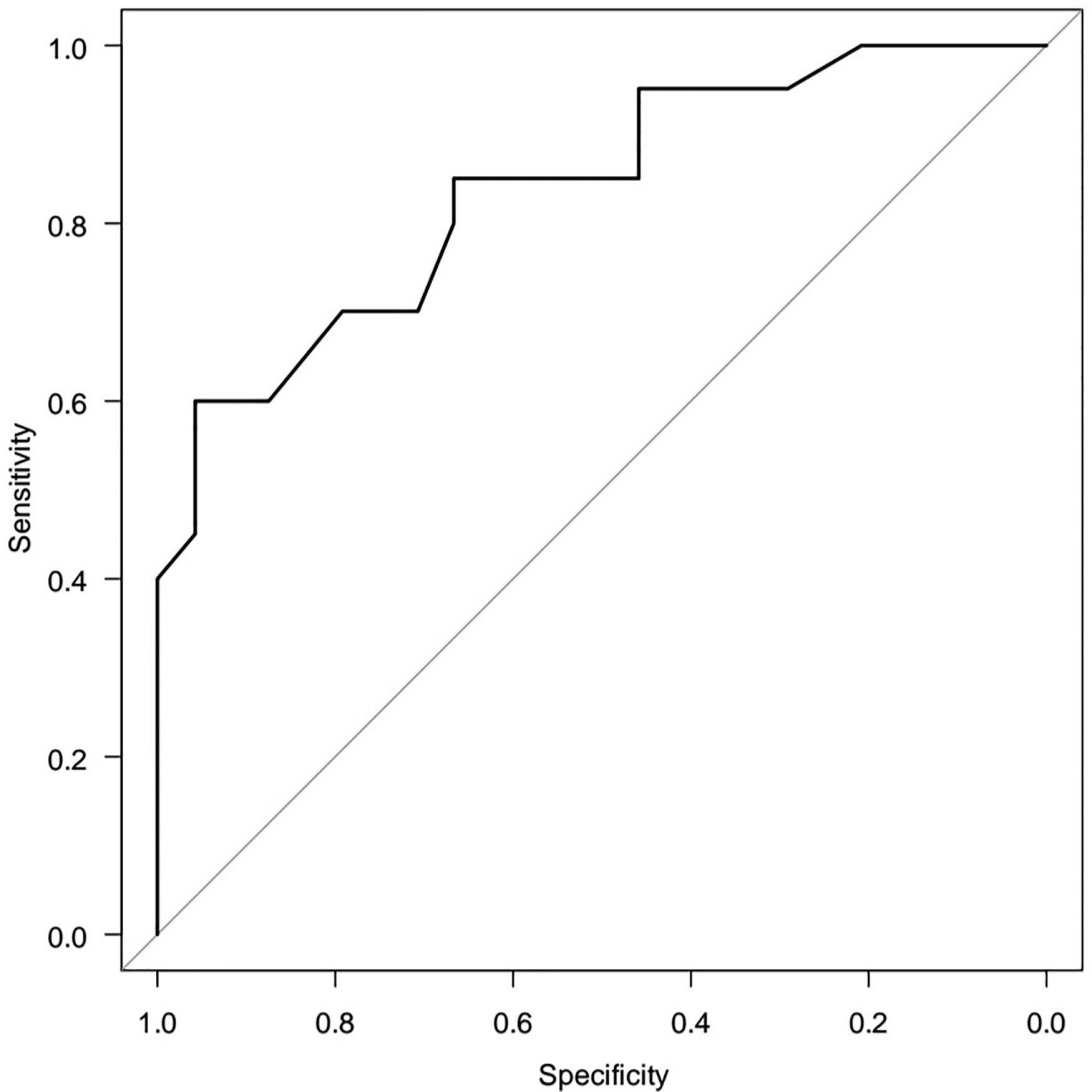
ROC-curve five-factor logistic regression model (AUC = 0.836 95% CI 0.717–0.956).

A five-factor model reveals the dependence of HOMA-2 IR (Y) on age (X_1_) and metabolic syndrome criteria (X_2_, X_3_, X_4_) the area under the ROC curve AUC = 0.836 (95% CI 0.717–0.956) is significantly (*p* < 0.05) greater than 0.5. To select a minimum set of factor features associated with objective variable, the method of step-by-step rejection/inclusion of explanatory variables (stepwise) was certified. Triglyceride level ≥1.7 mmol/L was statistically associated with the risk of high HOMA-2 IR [OR = 5.730 (95%CI 1.280–25.60) *p* = 0.0223] (Figure 6).

**Figure 7:**
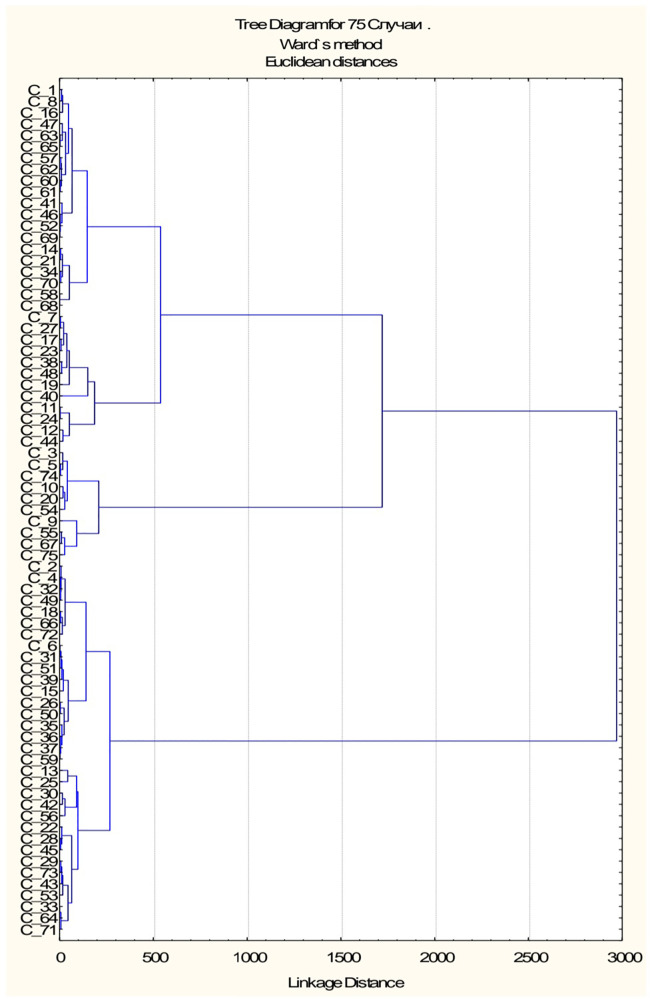
Ward's hierarchical dendrogram.

According parameters: HOMA-1 IR, HOMA-2 IR, %B and %S a dendrogram was constructed using Ward's hierarchical method, which clearly showed four clusters (Figure 7). We classified the resulting subgroups so that cluster 1 had the worst values of the studied parameters and cluster 4 had the best values (Table 1). By substituting anthropometric and lipid metabolic values into subgroups, a multiple comparison was made between the 4 samples. The *Kruskal-Wallis* one-way *analysis*-of-variance-by-*ranks* H-test of BMI (H = 4.1, *p* = 0.248) and WC (H = 3.9, *p* = 0.272) non-significant difference were obtained. It was found, that HOMA-1 IR was much higher in cluster 1 (6.32 ± 0.66) than in cluster 4 (2.19 ± 0.13). HOMA-2 IR was also much higher in cluster 1 (3.80 ± 0.34) than in cluster 4 (1.31 ± 0.06). By analysis of variance using Scheffe's multiple comparison method, a statistically significant difference was obtained between the laboratory parameters among the subgroups: HOMA-1 IR (*p* < 0.001), glucose (*p* < 0.001), insulin (*p* < 0.001), HOMA-2 IR (*p* < 0.001), %B (*p* < 0.001), %S (*p* < 0.001), TG (*p* = 0.005) and VLDL-C (*p* = 0.002). No statistically significant difference was found between other parameters of lipid metabolism: TC (*p* = 0.292), HDL-C (*p* = 0.213), LDL-C (*p* = 0.441), AC (*p* = 0.187) (Table 1).

**Table 1:** Characteristics of the studied patients by subgroups (clusters).

	Cluster 1	Cluster 2	Cluster 3	Cluster 4	^a^ *p*
	*n* = 20	*n* = 22	*n* = 18	*n* = 15	
Variables	Me ± m (Q1;Q3)	Me ± m (Q1;Q3)	Me ± m (Q1;Q3)	Me ± m (Q1;Q3)	
Age (y)	14 ± 0.36 (13;15)	15 ± 0.49 (14;16)	15 ± 0.52 (14;16)	15 ± 0.70 (11;15)	*p* = 0.086
BMI (kg/m²)	30.01 ± 1.25 (27.73;33.96)	27.89 ± 0.83 (26.56;30.82)	29.07 ± 1.07 (27.38;32.61)	27.45 ± 1.18 (24.68;30.56)	*p* = 0.248
WC (cm)	94 ± 2.36 (86;100)	90 ± 1.78 (88;92)	93.5 ± 2.03 (89;96)	90 ± 2.03 (86;92)	*p* = 0.272
	Mean ± SD (Min ÷ Max)	Mean ± SD (Min ÷ Max)	Mean ± SD (Min ÷ Max)	Mean ± SD (Min ÷ Max)	^b^ *p*
HOMA-1 IR	6.32 ± 0.66 (2.98 ÷ 9.06)	5.23 ± 0.38 (1.55 ÷ 12.7)	3.57 ± 0.22 (2.28 ÷ 5.67)	2.19 ± 0.13 (1.26 ÷ 3.15)	*p* < 0.001
Glucose (mmol/L)	4.28 ± 0.11 (4.19 ÷ 5.75)	4.89 ± 0.09 (3.28 ÷ 5.25)	5.15 ± 0.11 (4.4 ÷ 6.09)	4.78 ± 0.11 (3.88 ÷ 5.48)	*p* < 0.001
Insulin (mIU/mL)	32.24 ± 2.97 (15.6 ÷ 36.8)	23.75 ± 1.30 (10.62 ÷ 60.8)	15.76 ± 0.56 (12.4 ÷ 21.3)	10.27 ± 0.46 (6.62 ÷ 13.3)	*p* < 0.001
HOMA-2 IR	3.80 ± 0.34 (1.92 ± 4.65)	2.98 ± 0.17 (1.21 ± 6.71)	2.03 ± 0.08 (1.54 ± 2.82)	1.31 ± 0.06 (0.83 ± 1.74)	*p* < 0.001
%B	329 ± 14.38 (178.9 ± 235.6)	212.1 ± 3.77 (249.6 ± 483.1)	146.6 ± 3.38 (121.3 ± 169.7)	127.6 ± 4.99 (87.5 ± 159)	*p* < 0.001
%S	32.49 ± 3.76 (21.5 ± 52)	35.64 ± 1.99 (14.9 ± 82.5)	50.39 ± 1.87 (35.5 ± 64.8)	78.99 ± 4.32 (57.5 ± 121.1)	*p* < 0.001
TC (mmol/L)	3.97 ± 0.18 (2.83 ± 5.36)	3.73 ± 0.28 (3.06 ± 4.91)	3.62 ± 0.23 (2.42 ± 4.9)	4.29 ± 0.37 (2.1 ± 5.93)	*p* = 0.292
TG (mmol/L)	2.24 ± 0.39 (0.78 ± 3.5)	1.52 ± 0.29 (0.76 ± 6.16)	1.05 ± 0.14 (0.58 ± 1.95)	0.85 ± 0.09 (0.32 ± 1.21)	*p* = 0.005
HDL-C (mmol/L)	1.03 ± 0.04 (0.7 ± 1.66)	1.16 ± 0.09 (0.77 ± 1.33)	1.18 ± 0.08 (0.8 ± 1.9)	1.26 ± 0.09 (0.77 ± 1.77)	*p* = 0.213
LDL-C (mmol/L)	2.67 ± 0.16 (1.7 ± 3.96)	2.29 ± 0.26 (2.02 ± 3.71)	2.42 ± 0.21 (1.42 ± 3.72)	2.66 ± 0.32 (0.58 ± 4.1)	*p* = 0.441
VLDL-C (mmol/L)	0.82 ± 0.15 (0.34 ± 0.65)	0.45 ± 0.05 (0.35 ± 1.9)	0.37 ± 0.04 (0.27 ± 0.69)	0.34 ± 0.04 (0.15 ± 0.51)	*p* = 0.002
AC (IU)	3.09 ± 0.31 (1.45 ± 3.6)	2.26 ± 0.32 (1.62 ± 5.1)	2.31 ± 0.35 (1.24 ± 4.99)	2.15 ± 0.37 (0.35 ± 3.71)	*p* = 0.187

^a^ Kruskal-Wallis rank one-way analysis on ranks with multiple comparisons for 4 samples (clusters)

^b^ Analysis of variance. Scheffe's method of multiple comparisons for 4 samples (clusters)

## Discussion

In our study, there was a predominance of boys over girls, which confirms the results of a meta-analysis, where according to the IDF criteria MetS was more prevalent in males (3.46%; 95% CI 2.69, 4.23, I2 = 97.6%) than females (2.99%; 95% CI 2.34, 3.65, I2 = 95.6%) (2). The median of HOMA-1 IR was significantly higher than HOMA-2 IR, so by determining only the HOMA-1 IR we may lose some patients, on the other hand determining insulin resistance with HOMA-2 IR may be a more sensitive method and represent data from the current population. However, further studies of the MetS with a control group of healthy children are necessary. In a recent study, it was shown that HOMA-2 IR is more predictive than HOMA-1 IR for the progression to diabetes in pre-diabetes group, but there was no statistically significant difference in the non-diabetic group (16). An interesting phenomenon was that although males are more likely to suffer from MetS than females, they were found to have a higher insulin sensitivity. Further research is needed on gender differences in beta cell activity and insulin sensitivity in a larger sample of patients. Since there are no national reference values for HOMA-2 IR, we proposed level >2.26 to be the cut-off value for children and adolescents with MetS. However, the creation of national HOMA-2 IR percentile tables based on the age of child is important. It maybe emphasize that HOMA-2 IR was find to be even more sensitive for gender diferences than HOMA-1 IR. It was also also showen that TG level ≥1.7 mmol/L as one of the criteria of MetS was statistically associated with the risk of HOMA-2 IR >2.26. We found the correlations between HOMA-1 IR, HOMA-2 IR, %B and %S with TG and VLDL-C and a significant negative correlation between %B and HDL-C in childrens and adolescents with MetS. Similar results have been shown previously demonstrated by Shuang Zheng et al., in adults without diabetes (17). Surprisingly, by constructing a logistic model of regression, we found that the risk of high HOMA-2 IR decreases with age. This phenomenon requires further research.

Clustering of MetS in children and adults based on HOMA-1 IR, HOMA-2 IR, %B and %S on the basis of one pathological mechanism – insulin resistance, we conducted for the first time. Thus, we identified four subgroups at risk for the development of Mets complications. We recommend paying special attention to the first two subgroups, because they have poorer values not only of key indicators, but also to lipid metabolism. The first and the second clusters have the highest level of %B and the lowest level of %S. An abnormality in beta cell activity and loss of tissue sensitivity to insulin with decreased activity in peripheral tissues (mainly such as muscle, adipose and liver tissue) caused a number of metabolic and related diseases (18). A number of studies have shown that insulin resistance has been associated with a risk of developing asthma due to decrease glucose utilization and induction of abnormal fat metabolism in the skeletal muscles of respiratory system (19). A high prevalence of asthma was also found in children with high serum TG level (20). The elevated insulin can lead to increased androgen production, which can impair insulin sensitivity and lead to the metabolic imbalance that characterizes polycystic ovarian syndrome (PCOS) (21). Also, insulin resistance was associated with severity of obstructive sleep apnea (22). Based on data from earlier studies, it may be assumed that children in first two subgroups are more likely to have a high risk of developing comorbidities related to metabolic syndrome such as bronchial asthma, polycystic ovarian syndrome, behavioral disorders, obstructive sleep apnea and non-alcoholic fatty liver disease (23). So, the screening for these conditions should be recommended. All children of all subgroups we recommend to do HOMA-1 IR and HOMA-2 IR test with assessing *β*-cell function every 6 months.

## Conclusion

1. Median HOMA-1 IR was significantly higher than median HOMA-2 IR and that's why determinations insulin resistance only by HOMA-1 IR can ignore most patients.
2. We proposed HOMA-2 IR level >2.26 to be the cut-off value for children and adolescents with MetS.
3. Males had lower HOMA-2 IR and higher insulin sensitivity compared with females. No gender differences were found when comparing the HOMA-1 IR.
4. HOMA-1 IR, HOMA-2 IR, %B and %S were correlated with lipid metabolism parameters: TG and VLDL-C and negative correlated between %B and HDL-C in children and adolescents with MetS. The risk of getting a high TG result in the blood analysis in children with MetS was significantly associated with the HOMA-2 IR >2.26
5. Identification of four subgroups at risk of MetS complications in children and adolescentsis an effective tool in predicting the development of MetS in children and adolescents from mild to more severe and will help pediatricians to individualize their approach to the treatment of MetS.

## Data Availability

The original contributions presented in the study are included in the article/Supplementary Material, further inquiries can be directed to the corresponding author/s.
